# Changing environments during the Middle-Upper Palaeolithic transition in the eastern Cantabrian Region (Spain): direct evidence from stable isotope studies on ungulate bones

**DOI:** 10.1038/s41598-018-32493-0

**Published:** 2018-10-04

**Authors:** Jennifer R. Jones, Michael P. Richards, Lawrence G. Straus, Hazel Reade, Jesús Altuna, Koro Mariezkurrena, Ana B. Marín-Arroyo

**Affiliations:** 1 Instituto Internacional de Investigaciones Prehistóricas de Cantabria, (Universidad de Cantabria, Santander, Gobierno de Cantabria), Santander, 39005 Spain; 2 Department of Archaeology, School of Geosciences, University of Aberdeen, Aberdeen, AB24 3FX Scotland UK; 3 Simon Fraser University, Department of Archaeology, Burnaby, V5A 1S6 B.C Canada; 4 University of New Mexico, Anthropology Department, MSC01 1040, Albuquerque, NM 87131 USA; 5 UCL Institute of Archaeology, 31-34 Gordon Square, London, WC1H 0PY UK UK; 6 Centro de Conservación e Investigación de los Materiales Arqueológicos y Paleontológicos de Gipuzkoa, Paseo Zarategi, 84-88, Donostia/San Sebastián, 20015 Spain; 7 Leverhulme Centre for Evolutionary Studies, Department of Archaeology and Anthropology. University of Cambridge, Cambridge, CB2 1QH United Kingdom

## Abstract

Environmental change has been proposed as a factor that contributed to the extinction of the Neanderthals in Europe during MIS3. Currently, the different local environmental conditions experienced at the time when Anatomically Modern Humans (AMH) met Neanderthals are not well known. In the Western Pyrenees, particularly, in the eastern end of the Cantabrian coast of the Iberian Peninsula, extensive evidence of Neanderthal and subsequent AMH activity exists, making it an ideal area in which to explore the palaeoenvironments experienced and resources exploited by both human species during the Middle to Upper Palaeolithic transition. Red deer and horse were analysed using bone collagen stable isotope analysis to reconstruct environmental conditions across the transition. A shift in the ecological niche of horses after the Mousterian demonstrates a change in environment, towards more open vegetation, linked to wider climatic change. In the Mousterian, Aurignacian and Gravettian, high inter-individual nitrogen ranges were observed in both herbivores. This could indicate that these individuals were procured from areas isotopically different in nitrogen. Differences in sulphur values between sites suggest some variability in the hunting locations exploited, reflecting the human use of different parts of the landscape. An alternative and complementary explanation proposed is that there were climatic fluctuations within the time of formation of these archaeological levels, as observed in pollen, marine and ice cores.

## Introduction

Marine Isotope stage 3 (MIS3) (60-25ka BP) was a period of instability with rapid and acute climatic changes^1,2^. Mid-late MIS3 was the time of the Middle-Upper Palaeolithic transition (c.45-25ka BP) (MP-UP), when late Neanderthal populations became extinct and were replaced by Anatomically Modern Humans (AMH). Neanderthal extinction is now known to have been relatively rapid, following a regional pattern, rather than a uniform pan-European one^3^. Climate cannot be claimed as a homogeneous, monolithic driver for their extinction in a single event across the continent as previously proposed by some^4–7^. How climate was expressed locally within the continental scale of Europe is not well understood, with climatic proxies identified from caves such as pollen, charcoal and plant remains, as well as microstratigraphies for this period all being relatively scarce, and although useful as environmental indicators^8^, they can be subject to taphonomic and diagenetic alterations^9,10^. There is a lack of radiometric chronologies for environmental and archaeological records independent of ice^11^ and marine cores^12,13^, which are not directly related to the localised conditions experienced at the archaeological sites. New methods have been recently proposed with great promise to overpass these limitations for a continental scale such as tephrochronology^14^ or atmospheric circulation modelling^15^, but they have some limitations and higher levels of precision are required.

Since the early 2000s, δ^13^C and δ^15^N analyses have been used to reconstruct animal palaeoecology and past environments^16^. Bone collagen δ^13^C and δ^15^N analysis within animal bones derive directly from diet consumed^17^, representing long-term feeding behaviour, informing on average environmental conditions throughout the period of bone growth^18,19^. Collagen δ^34^S analysis can be used as a locational tool, with values directly linked to local geology and soil type, proximity to the sea and rainfall^20^. These techniques have successfully been applied to European Palaeolithic environmental reconstructions directly related with human occupations to unravel the ecological conditions those populations confronted^16,21–30^.

**Figure 1:**
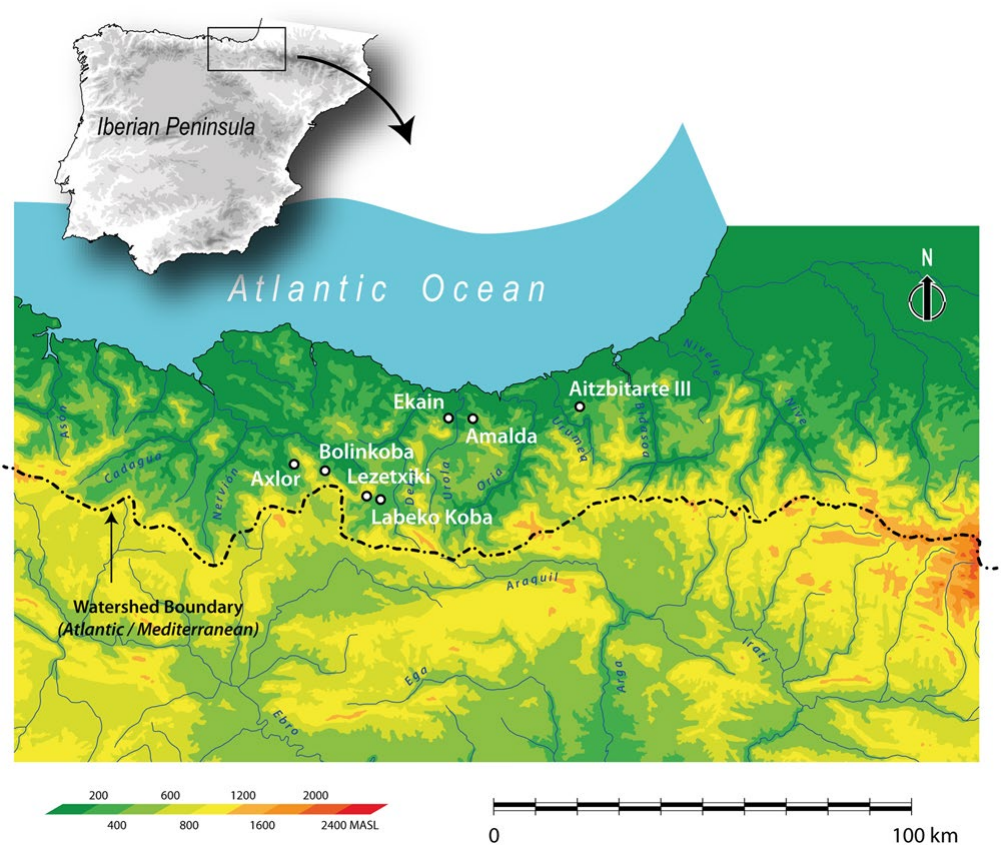
Map of the eastern Cantabrian region, northern Spain, showing the locations of the Bizkaia and Gipuzkoa sites studied. In Bizkaia: Axlor and Bolinkoba. In Gipuzkoa: Lezetxiki, Labeko Koba, Ekain, Amalda and Aitzbitarte III.

An ideal location to apply this methodology to reconstruct the conditions faced by late Neanderthals and early AMH during MIS3 is the Cantabrian region in the Atlantic zone of northern Spain, which contains a high density of Middle and early Upper Palaeolithic sites^31^. The Cantabrian geographical region is formed by the Autonomous Communities of Asturias in the west, Cantabria in the centre and the Basque Country in the east. In this paper, we focus on the latter sector which is encompassed by the modern-day provinces of Gizpukoa, Bizkaia and Alava (Fig. 1). Gipuzkoa and Bizkaia are ecologically distinct from the western sectors of the Cantabrian region and are characterised by deep, steep-sided closed in mountains that often drop directly to the ocean. The Cantabrian Cordillera mountain range, relatively low in the Basque sector, separates the Atlantic coastal region from the Mediterranean-draining Ebro basin^32^. A recent chronological review of dates for MP-UP transitional sites undertaken using ultrafiltration radiocarbon method has provided a high-precision sequence of events for the timing of both human species’ activities in the region^3,33–36^. Therefore, it is now possible to obtain an accurate, independently dated environmental record in those archaeological sites by undertaking stable isotope analysis of ungulate bones exhibiting evidence of human manipulation (i.e. cut marks and fresh fractures) during the Mousterian, Châtelperronian, Aurignacian and Gravettian periods. The results can be integrated with the available sedimentology, palynological and micro and macromammal data. Results of bone collagen δ^13^C, δ^15^N and δ^34^S analyses on macromammals have identified temporal, intra- and inter-site trends in the climatic and environmental conditions directly experienced by late Neanderthals and early AMH in this archaeologically important region.

## Sites and Materials

### Geographical setting of the region

The sites analysed in this article are located in the two small coastal provinces of the Spanish Basque Country (the autonomous region of Euskadi): Gipuzkoa and Bizkaia. This is an eminently mountainous area straddling 43°10′ north latitude, bounded to the north by the Bay of Biscay, to the south by the Basque Mountain ranges of the Cantabrian Cordillera, to the east by the western end of the Pyrenees and to the west by the orographically less “chaotic”/structurally “banded” regions of Cantabria and Asturias. The coastal Basque provinces are connected to the SW corner of France via a very low pass between Monte Jaizkibel and Mont Rhune at the Bidasoa River and to the interior, upland Basque province of Alava and the autonomous region of Navarra via the lowest (600–900 m a.s.l.) passes across the entire Cantabrian Cordillera^37^. During the Last Glacial, there were mountain glaciers on some of the highest peaks of the Basque Mountains (e.g., Gorbea 1475 m a.s.l.; Aralar 1300 m a.s.l.), but with no continuous ice sheet, access to the semi-distant flint sources to the south of the Cordillera in Alava, Navarra and Treviño was always possible. The region is generally characterized by deep, narrow, short valleys, separated by high mountain ridges that often extend all the way to the shore. Under full glacial conditions, only a very narrow band of the inner continental shelf was exposed, but enough to provide what was probably the easiest east-west avenue of communication across the region and between it and the French Basque Country and Cantabria, respectively. Today characterized by a temperate, oceanic, pluvial climate (heavily dominated by the Gulf Stream), this region was probably always relatively humid even under the rigorously cold conditions of the Last Glacial (when the Gulf Stream was absent). In sharp contrast to the Mediterranean environments of the upper Ebro Basin to the south of the Cordillera, the Basque Country is in the Eurosiberian ecological zone, and differences (albeit attenuated) also existed during the Last Glacial.

### Sites sampled

The eastern sector of the Cantabrian region contains several key sites pertaining to the late Mousterian, Châtelperronian, Aurignacian and Gravettian technocomplexes including Bolinkoba and Axlor in Bizkaia and Labeko Koba, Amalda, Aitzbitarte III, Lezetxiki and Ekain in Gipuzkoa (Table 1). Although there are other contemporaneous sites in those provinces, the sites analysed were selected because they are considered key regional sites with well-established stratigraphies that have been reviewed and recently dated by ultrafiltration^33–36^ (Table 2). Additionally, these sites have been the subject of technological studies and some taphonomic analyses that proved human presence^38–43^ and, also have environmental datasets^44–49^. Sites and levels sampled are presented in Table 1, with AMS radiocarbon dates shown in Table 2. Briefly, we describe the sites and levels included in this study and their chronology, from west (Bizkaia) to east (Gipuzkoa).

**Table 1:** Summary of the specimens sampled per sites and archaeological levels and their chrono-cultural adscription.

Broad cultural attribution	Temporal span (Ka BP uncal).	Site	Level	No. Samples for δ^13^C and δ^15^N analysis		No. aliquots analysed for δ^34^S	
				Red deer	Horse	Red deer	Horse
Gravettian	28	Bolinkoba	VI/F	4	7	—	—
		Amalda	VI	5	6	1	3
	31–29	Aitzbitarte III	IV	5	—	2	—
			Va	5	—	4	—
			Vb Upper	1	2	1	1
Aurignacian	31	Ekain	IXb	4	—	—	—
		Aitzbitarte III	Vb Central	5	2	2	1
	35–32	Labeko Koba	IV	—	1	—	—
			V	5	5	2	2
			VI	5	4	3	—
Châtelperronian	38–37		IX Lower	5	6	2	2
Mousterian	n/a	Lezetxiki	V	4	—	—	—
			IV	5	—	—	—
	44–42	Amalda	VII	5	5	1	3
	>49	Axlor	IV	12	2	—	—
	60–50 (based on typology)		VI	12	6	—	—
			VIII	10	—	—	—
**Totals**				**92**	**46**	**18**	**12**

**Table 2:** Radiocarbon dates from sites and levels sampled, with laboratory codes and sample preparation methods used. AF: collagen extracted using the ultrafiltration method prior to analysis. Bone sample code is included for bones, that were both dated and analysed for stable isotopes.

Culturalperiod	Site	Level	Radiocarbon date	Lab code	Dating method	Bone Sample code	Ref.
Gravettian	Bolinkoba	IV/F	25280 ± 210	OxA-32519	AF	BOL03	^33^
			29,950 ± 120	Beta-426854	AMS	—	^59^
			21,020 ± 90	Beta-302981	AMS	—	^59^
	Amalda	VI	27400 ± 1000	I-11–665	C14	—	^74^
			27400 ± 1100	I-11-664	C14	—	^74^
			28540 ± 310	OxA-32426	AMS AF	AMA26	^33^
			28710 ± 300	OxA-34934	AMS AF	AMA25	^33^
	Aitzbitarte III	IV	28,320 ± 605	Ua-18465	AMS	—	^136^
			22,420 ± 290	Ua-24965	AMS	—	^136^
			27,580 ± 550	Ua-18464	AMS	—	^136^
			26,260 ± 480	Ua-37961	AMS	—	^136^
			24,240 ± 365	Ua-11146	AMS	—	^136^
			25,815 ± 475	Ua-11148	AMS	—	^136^
			29,130 ± 310	OxA-32422	AMS AF	AIT26	^33^
			29,020 ± 320	OxA-32499	AMS AF	AIT13	^33^
		Va	31,210 ± 860	Ua-18467	AMS	—	^136^
			28,950 ± 655	Ua-18466	AMS	—	^136^
			28,530 ± 645	Ua-37962	AMS	—	^136^
			27,165 ± 520	Ua-24966	AMS	—	^136^
			26,350 ± 475	Ua-24967	AMS	—	^136^
			31,090 ± 400	OxA-32420	AMS AF	AIT7	^33^
			31,300 ± 400	OxA-32421	AMS AF	AIT10	^33^
		Vb Upper	31,950 ± 450	OxA-32419	AMS AF	AIT21	^33^
			30,990 ± 390	OxA-32416	AMS AF	AIT2	^33^
Aurignacian		Vb Central	31,130 ± 390	OxA-34932	AMS AF	Only dated	^33^
			31,600 ± 400	OxA-32418	AMS AF	AIT19	^33^
			34,900 ± 400	OxA-32417	AMS AF	AIT05	^33^
	Ekain	IXb	>30,600	I-11506	C14	—	^70^
			31,140 ± 400	OxA-32423	AMS AF	EK02	^33^
			31,110 ± 400	OxA-32424	AMS AF	EKA05	^33^
	Labeko Koba	Level IV	33,550 ± 550	OxA-21780	AMS AF	—	^35^
			33,600 ± 500	OxA-21768	AMS AF	—	^35^
		Level V	34,750 ± 600	OxA-21767	AMS AF	—	^35^
			34,650 ± 600	OxA-21779	AMS AF	—	^35^
		Level VI	32,200 ± 450	OxA-21794	AMS AF	—	^35^
			32,150 ± 450	OxA-21841	AMS AF	—	^35^
			35,100 ± 600	OxA-21778	AMS AF	—	^35^
Châtelperronian	Labeko Koba	IX Lower	37,900 ± 900	OxA-22564	AMS AF	—	^35^
			37,400 ± 800	OxA-22506	AMS AF	—	^35^
			38,000 ± 900	OxA-22561	AMS AF	—	^35^
			38,100 ± 900	OxA-22562	AMS AF	—	^35^
			37,800 ± 900	Oxa-22563	AMS AF	—	^35^
Mousterian	Amalda	VII	44,500 ± 2100	OxA-32500	AMS AF	AMA2	^35^
			42,600 ± 1600	OxA-34933	AMS AF	AMA5	^35^
	Axlor	IV	42,010 ± 1280	Beta-144262	AMS	—	^54^
			>43,000	Beta-22586	AMS	—	^54^
			>49,300	OxA-32428	AMS AF	AXL39	^33^
			>49,900	OxA-32429	AMS AF	AXL42	^33^

Axlor in Dima (Bizkaia) is a rock-shelter that was excavated by J. M. Barandiarán between 1967 and 1974, revealing a long sequence of Mousterian Levels I-VIII^50,51^. Subsequent interventions between 1999 and 2004 linked a detailed stratigraphy (Levels B-N) to the levels recorded by Barandiarán^52,53^. In this study, Levels VIII, VI and IV from the Barandiarán’s excavations were analysed, and correlated to new Levels N, M and D, respectively^38^. New ultrafiltration dates were available for the uppermost of these levels, Level D, but were beyond the radiocarbon limit (>49,300 OxA-32428 and >49,900 OxA-32429). These dates are considerably older than previous ones achieved for this level using AMS but without ultrafiltration: 42,010 ± 1,280 BP (Beta-144,262) and >43,000 (Beta-22586)^37,54,55^. These new results suggest that the lower levels at Axlor are significantly older than previously thought. Based on artefact typology and technology, they are now estimated to date to around 60–50ka BP^38^. Macromammals represented are red deer, followed by Spanish ibex and large mammals (horse and bovines), with evidence of butchering marks and fresh breakage patterns^50,51,56,57^. Although a complete taphonomic analyses of the complete assemblage has not been undertaken, a recent the review of carnivore and bird bones have revealed evidence of manipulation by Neanderthals^38^ (Table 3)

**Table 3:** Macromammal faunal assemblage NISP values represented in the levels of the archaeological sites sampled in this study, indicating when available references of taphonomic studies.

Site and Level	Axlor			Amalda	Labeko Koba				Ekain	Aitzbitarte III				Bolinkoba	Amalda	NISP total per cultural periods			
	VIII	VI+V	IV	VII	IX lower	VI	V	IV	IXb	Vb central	Vb upper	Va	IV	VI/F	VI				
Cultural attribution	Mousterian				Châtelperronian	Aurignacian					Gravettian					Moust.	Châtel.	Aurig.	Gravet.
*Mammuth primigenius*	—	—	—	—	—	—	6	5	—	—	—	—	—	—	0	—	—	11	—
*Equus ferus*	3	23	64	48	210	36	61	53	—	12	—	—	2	—	101	138	210	162	103
*Bovini*	2	61	160	58	143	175	290	161	21	51	53	105	180	1	99	281	143	698	438
*Capra pyrenaica*	28	117	179	61	—	—	—		—	—	—	—	—	9	236	385	—	—	245
*Rupicapra rupicapra*	13	75	10	536	—	4	7	8	133	22	9	44	88	1	2769	634	—	174	2911
*Megaloceros giganteus*	—	—	—	—	—	—	—	1	—	—	—	—	0	0	0	0	—	1	—
*Cervus elaphus*	120	292	127	150	792	59	29	13	26	71	65	53	72	2	274	689	792	198	466
*Rangifer tarandus*	0	0	7	0	14	1	—	1	—	—	—	4	2	0	2	7	14	2	8
*Capreolus capreolus*	1	1	0	3	0	—	—	1	6	3	—	8	5	0	17	5	—	10	30
*Sus scrofa*	1	1	0	0	0	—	—	—	—	—	—	0	0	0	3	2	—	—	3
Sub-Total	168	570	547	856	1159	275	393	243	186	159	127	214	349	13	3501	2141	1159	1256	4204
*Canis lupus*	—	3	1	17	—	2	1	—	2	—	—	2	2	—	37	21	—	5	41
*Vulpes vulpes*	—	—	3	29	2	67	6	16	10	—	4	4	—	—	133	32	2	99	141
*Alopex lagopus*	—	—	—	—	—	—	—	—	—	5	—	—	23	—	1	—	—	5	24
*Cuon alpinus*	—	—	—	1	—	—	—	—	—	—	—	—	—	—	1	1	—	—	1
*Ursus spelaeus*	1	1	3	58	3	20	—	4	29	—	—	10	7	—	103	63	3	53	120
*Meles meles*	—	—	1	—	—	—	—	—	—	—	—	—	—	—	—	1	—	—	—
*Mustela erminea*	—	—	—	—	—	—	—	—	—	—	—	1	5	—	—	—	—	—	6
*Mustela nivalis*	—	—	—	—	—	—	—	—	—	8	8	5	21	—	—	—	—	8	34
*Mustela putorius*	—	—	—	—	—	—	—	—	—	—	—	—	1	—	—	—	—	—	1
*Crocuta crocuta*	—	—	—	3	55	43	2	9	0	2	1	—	1	—	11	3	55	56	13
*Panthera leo*	—	—	—	—	—	—	—	—	—	—	—	1	—	—	—	—	—	—	1
*Panthera pardus*	—	—	—	3	—	—	—	—	—	—	—	—	—	1	3	3	—	—	4
*Lynx*	—	1	—	—	—	—	—	—	—	—	—	—	1	—	—	1	—	—	1
*Felis silvestris*	—	—	—	—	—	—	1	—	—	—	—	—	1	—	—	—	—	1	1
*Marmota marmota*	1	1	—	—	—	—	—	—	—	—	—	—		—	—	2	—	—	—
Sub-Total	2	6	8	111	60	132	10	29	41	—	13	23	62	1	289	127	60	227	388
TOTAL	170	576	555	967	1219	407	403	272	227	174	140	237	411	14	3790	2268	1219	1483	4592
Taphonomic analysis	NO			YES	YES				YES	YES				NO	YES				
References	^49, 54, 55^			^40, 41, 138^	^65, 81^				^53, 69^	^42^				^58^	^40, 41, 71^				

Bolinkoba, in Abadiño (Bizkaia), was originally explored by J.M. Barandairán and T. Aranzadi in the early 1930s^58^, followed by excavations by J.M. de Urquijo in the 1940s and again recently, between 2008 and 2014 by M.J. Iriarte^59^. The site stratigraphy reveals a series of levels from the Gravettian to the Azilian. Pertinent to this project, Gravettian Level VI (F) from the Barandiarán excavations was selected and a new date indicates a Gravettian occupation (25,280 ± 210 OxA-32519)^33^, although AMS dates from new excavation have provided dates of 29,950 ± 120 (Beta-426854) and 21,020 ± 90 (Beta-302981) for the same level^59^ (Iriarte and Arrizabalaga 2015). Recent review of macromammal remains from the Barandiaran and Aranzadi collection and, from the recent excavations, indicate similar proportions and presence of ungulates between both assemblages, with Spanish ibex being the most common hunted species followed by red deer^60^ (Table 3). Despite the small representation of micromammals, these show cold palaeoenvironmental conditions during the Gravettian that included a modest decline in forest^61^. Palynological results did not provide palaeoenvironmental information due to the poor spore and pollen preservation^62^ (Table 4).

**Table 4:** Summary of available environmental proxies for the studied sites, including references for the information provided. Dashes denote levels where datasets are missing.

Broad cultural attribution	Temporal span (Ka uncal BP)	Site	Level	Pollen	Sediment interpretations	Macrofauna	Microfauna
Gravettian	28	Bolinkoba	VI/F	—	—	—	—
		Amalda	VI	*Pinus* accounts for around 10% of pollen, with *Betula* present. *Poaceae/Graminae* grasses are common (although varying between 5-40% in samples), and *Anthemidae* and *Cichorieae* are represented. More temperate *Corylus* accounts for 15–20% of pollen^76^.	Cool/wet environment^137^	Total ungulate NISP = 3501, chamois 79%, red deer 8%, spanish ibex 7%, bos/bison 3%, and horse 3%. Presence of cool indicator species reindeer and arctic fox^138^	Total NISP = 490, dominance of *Microtus* *arvalis-agrestis* (74%). Cold adapted *Microtus oeconomus* present in smaller numbers than previously seen in the sequence (7%), and presence of *P. lenki* (>1%). *Pitymys* sp. (5%). Increased frequency of species that thrive in moist/temperate conditions including *Sorex araneus* (7%), *A. terrestris* (5%), *Talpa europea* and *N. fodiens also* present^139^
	31–29	Aitzbitarte III	IV	Low percentage of tree pollen 2.5% (*Pinus* declines slightly and *Cupressaceae* increases). *Poaceae* declines (9.5%) and an increase in the composites is seen (except *Cedrela tubiflora*). Spores decrease (3.5%)^44^	Slightly warmer climate compared to previous parts of the sequence^140^.	Total ungulate NISP = 354, bos/bison 52%, chamois 25%, red deer 12%, horse, roe deer and reindeer all present^43^	Total NISP = 1471, *Microtus* *arvalis-agrestis* common (47%), and *Microtus oeconomus* well represented (17%). *Sorex araneus* (12%), *Pitymys* sp. (5%) and *Talpa* sp. (12%), also identified, alongside *A. terrestris* (4%). Presence of *Neomys* sp. and *Sorex minutus*^79^.
			Va	Tree cover remains stable. Steppic vegetation including grasses *Poaceae/Gramineae* (25%), umbelliferous plants (6%) and *rosaceae* (2%) present. *Cedrela tubiflora* and *Compositae liguliflor* (31%) common as seen previously. Spores up to 8%^44^	Conditions expressed were more severe^79^	Total ungulate NISP = 211, bos/bison 49%, red deer 25%, chamois 20%, roe deer and reindeer also present^43^	Total NISP = 1155, *Microtus arvalis-agrestis* common (51%) and *Microtus oeconomus* well represented (17%). *Sorex araneus* (15%), *Pitymys* sp. (7%) and *Talpa* sp. (6%) also identified, alongside *A. terrestris* (3%). *Sorex minutus* and *Apodemus* sp. present^79^.
			Vb upper	Slight recovery in tree cover (up to 2.5%- *Pinus* and *Cupressaceae*) Steppic vegetation dominates, *Poaceae* starts to increase*. Centaurea* declines to 8%, *Compositae liguliflor* accounts for 41% pollen. Level generally similar in composition to levels Vb Central and Va^44^	Slightly cooler conditions were experienced at this time^140^	Total ungulate NISP = 127, red deer 51%, bos/bison 42%, chamois 7%^43^	—
Aurignacian	31	Ekain	IXb	—	—	Total ungulate NISP = 398, more temperate suite of fauna: chamois 65%, bos/bison 19%, red deer 15%^72^	Total NISP = 31, *Arvicola* most common species (NISP = 27), *Pliomys lenki* and *Pitymys* sp. also present^73^
		Aitzbitarte III	Vb Central	1.6% tree cover, pine and juniper. Steppic vegetation dominates with of *Centaurea* common (32–35%) and *Compositae,* in addition to *Cedrela tubiflora* and *Compositae liguliflor* found in variable proportions within the samples^44^	Possibly slightly cooler conditions experienced	Total ungulate NISP = 158, red deer 44%, bos/bison 32%, chamois 14%, horse 8%, and roe deer also present^43^.	Total NISP = 574, *Microtus* *arvalis-agrestis* common (51%), and *Microtus oeconomus* present in substantial quantities (15%). *Sorex araneus* (14%), *Pitymys* sp. (5%) and *Talpa* sp. (8%) also represented, alongside *A. terrestris* (7%)^79^.
	35–32	Labeko Koba	IV	—	Conditions still cool, but indicate the start of a tempering environment^141^	Total ungulate NISP = 237, bos/bison 68%, horse 22%, red deer 5%, chamois 3%, and reindeer present^85^	Total NISP = 40. *Arvicola terrestris* (NISP = 38) and *Talpa* sp. (NISP = 2) present^77^.
			V	Pollen evidence sparse. *Pinus* present (15%) alongside steppic vegetation, such as *Cedrela tubiflora* (8%), *Compositae liguliflor* (25%) and *Poaceae/Gramineae* (15%)^45^	Cold conditions remaining in this level^141^	Total ungulate NISP = 387, bos/bison 75%, horse 16%, red deer 7%, and chamois 2%)^85^	Total NISP = 74. *Arvicola terrestris* most common species (NISP 67) *Microtus agrestris*-*arvalis*, *Talpa* sp. *Glis glis* and present^77^.
			VI	Climatic conditions interpreted as declining, decrease in the mid-thermophilic taxa observed including *Poaceae/Gramineae* (40–50%), *Plantago* (c. 10%), *Cedrela tubiflora* and *Compositae liguliflor* (both representing c.10% of samples taken). Low quantities of arboreal pollen, mostly *Pinus* (10–15%) persist^45^	—	Total ungulate NISP = 275 bos/bison 64%, red deer 21%, horse 13%, and chamois present^85^	Total NISP = 43. *Arvicola terrestris* (NISP = 37) most common, *Talpa* sp., *Microtus agrestris-arvalis* present^77^
Châtelperronian	38–37	Labeko Koba	IX Lower	Conditions fairly benign. Presence of mid-thermophilic species such as *Corylus* (5–7%). Increased evidence of steppic vegetation (60% in one sampled) *Pinus* also present (c.12–15%)^45^	—	Total ungulate NISP = 1159, red deer 68%, horse 18%, bos/bison 12%, and presence of reindeer^85^	Total NISP = 50. *Arvicola terrestris* (NISP = 40) most common. *Talpa* sp. *Glis glis, Microtus agrestris-arvalis* present^77^
Mousterian	44–42	Amalda	VII	Poor pollen preservation in this level, represented predominantly by *Pinus* (c.60%), with *Cichorioideae* accounting for c. 20% of total pollen and low frequencies of *Chenopodiaceae*^76^.	—	Total ungulate NISP = 856, chamois 63%, red deer 18%, bos/bison 7%, spanish ibex 7% and horse 6%^138^	Total NISP = 22. *Microtus arvalis- agrestis* (NISP = 14) most common. Cool indicator species *Pliomys lenki* present in addition to *A. terrestris, Apodemus* sp., and *Sorex*^139^
	>49	Axlor	IV	—	—	Total ungulate NIS = 109 Red deer 44%, bos/bison 30%, Spanish ibex 15%, horse 7%, and chamois 4%^56,142^	—
			VI	—	—	Total ungulate NISP = 585, bos/bison 15%, spanish ibex 15%, red deer 14%, horse 6% and chamois present^56,142^	—
	60–50		VIII	—	—	Total ungulate NISP 163, red deer 74%, spanish ibex 14%, chamois 8%, horse 2%, and presence of bos/bison, roe deer and wild boar^56,142^	—

Labeko Koba (Arrasate, Gipuzkoa) was discovered during the construction of a road and was the object of a programmed salvage excavation during 1987–88. The now-destroyed cave held one of the few Châtelperronian levels (Level IX lower) together with Morin Level 10 and Aranbaltza, in the entire Cantabrian region^35,63,64^. There is a lack of human remains in sites attributed to the Cantabrian Châtelperronian, although recent research has suggested that this techno-complex was the work of Neanderthals^33^. The Labeko Châtelperronian is followed by Early Upper Palaeolithic levels, with a sequence of Protoaurignacian (Level VII) and Early Aurignacian technocomplexes (Levels IV-VI)^65–67^. These have been recently dated, producing an accurate chronology of the modern human occupation at the site^35^. The archaeozoological and taphonomic analyses revealed that the site was alternatively used as an occasional hunting camp and a carnivore den^68,69^ (Table 3). Levels sampled for stable isotope analysis were IX lower (Châtelperronian) and Early Aurignacian Levels VI, V and IV. No well-preserved faunal remains were available from Level VII and Level IX upper was not sampled as it was archaeologically sterile^35,66^. Palynological remains from Level IX lower reveal the presence of mesothermophilic species, including *Castanea*, while the other levels display characteristically stadial botanical associations, with a low representation of arboreal taxa^45^ (Table 4).

Ekain, located in Deba (Gipuzkoa), has a stratigraphic sequence ranging from the Early Upper Palaeolithic through the Upper Magdalenian and Azilian, and is well-known for its late Upper Palaeolithic cave art^70^. The lower part of the sequence contains evidence of human presence during the Initial Upper Palaeolithic. Relevant to this study, Level IXb was interpreted as an Early Aurignacian sporadic camp with a small lithic assemblage, probably accumulated in no more than a single occupation episode^71^. Level IXb was dated by conventional ^14^C to >30,600 (I-11506) but recent AMS ultrafiltered dates of 31,140 ± 400 (OxA-32423) and 31,110 ± 400 (OxA-32424) show that this level is contemporaneous with the Evolved Aurignacian from Aitzbitarte III (entrance) Vb central (Gipuzkoa) and La Viña Levels XIII and XII (Asturias)^33^. *Ursus spelaeus* is abundant in this level (48%), but this is a lower relative frequency than in Level Xa (91%). Other carnivores are also present, including: fox, wolf, hyena and panther. Ungulates such as bovines, chamois and red deer are represented in the assemblage and have been interpreted as occasional elements of human diet^72^ (Table 3). Within the micromammal assemblage *Arvicola* sp. has been identified^73^ (Table 4).

Amalda in Zestoa (Gipuzkoa) is known for its succession of Mousterian and Gravettian levels, as well as Late Upper Palaeolithic occupations^74^. Mousterian Level VII was once interpreted to have been an anthropogenic occupation level. Subsequent, taphonomic analysis proposed that this level was the deposit of a carnivore den visited sporadically by Neanderthals^40,41^. However, recent reanalysis reaffirmed that the level’s contents were mainly human-derived, but with occasional carnivore activity^42,74,75^ (Table 3). Recent AMS ultrafiltered dates of 44,500 ± 2100 (OxA-32500) and 42,600 ± 1600 (OxA-34933) confirm its Mousterian chronology^33^. The Gravettian technocomplex is present in Level VI, which is dated to between 28,540 ± 310 (OxA-32426) and 28,710 ± 300 (OxA-34934). All animal samples analysed from this site were selected from areas where stratigraphic integrity remained intact. Despite poor pollen preservation, the spectrum from Level VII is represented predominantly by pine trees (c.60%), with *Cichorioideae* accounting for c. 20% of total pollen and low frequencies of *Chenopodiaceae*. There are more temperate pollen species in Level VI including *Corylus* and *Betula*^76^ (Dupré 1990). The scarce micromammal remains recovered in Level VII are from *Microtus arvalis-agrestis* and *Pliomys lenki*, *Arvicola terrestris*, *Apodemus* sp. and *Sorex* and include cool climate indicator species. In Level VI the representation of cold adapted species is lower and there is an increased frequency of species that thrive in moist/temperate conditions including *Sorex araneus* (7%), *A. terrestris* (5%), with *Talpa europea* and *N. fodiens* also present^77^ (Table 4).

Aitzbitarte III is part of a karstic system with five different caves in Errenteria, Gipuzkoa, near San Sebastián. The most recent excavations and analyses of material from the site were undertaken in the entrance area of the cave between 1994 and 2002, revealing a cultural sequence of Evolved Aurignacian (Level Vb central) and Gravettian (Levels Vb upper, Va and IV)^78^. Ultrafiltration dates reveal a rapid transition from the Aurignacian to the Gravettian. The dates at the cave entrance show that these levels represent a short span of time and were formed of discrete periods of occupation. The dates for Level Vb Central are 31,130 ± 390 (OxA-34932) and 31,600 ± 400 (OxA-32418)^33^ which are coherent with previous AMS dates^78^ and the cultural attribution to an Evolved Aurignacian phase. Dates obtained from Level Vb upper of 31,950 ± 450 (OxA-32419) and 30,990 ± 390 (OxA-32416) are similar to those obtained in Level Vb Central, indicating either an Evolved Aurignacian occupation that already shows some features transitional to the Gravettian or the first manifestation of an Early Gravettian at the site (Rios-Garaizar *et al*. 2013). New dates in Level Va, previously identified as Early Gravettian with Noailles burins^33,71^, appear to support a quick transition towards the Gravettian, with both dates being very similar: 31,300 ± 400 (OxA-32421) and 31,090 ± 400 (OxA-32420), and are consistent with other dates obtained in Levels Vb Central and Vb Upper. Finally, for Level IV, also classified as Early Gravettian with Noailles burins, the new dates of 29,130 ± 310 (OxA-32422) and 29,020 ± 320 (OxA-32499) are consistent with other Early Gravettian dates in the region. Archaeozoological and taphonomic analyses indicate an intense exploitation of red deer and bovines, followed by chamois and horse during the Aurignacian, while during the Gravettian bovines are the most common taxa, followed by red deer, chamois and horse^43^ (Table 3). Micromammals have a similar faunal composition during both periods; *Microtus agrestis-arvalis* is the dominant group and *Pliomys lenki* is sparsely found. These species reflect harsh climatic conditions, cold and humid, with open landscapes and scarce woodlands^79^. Pollen also reflect cold conditions with variations in the moisture, that indicate a landscape dominated by herbaceous-shrub taxa, with very little representation of trees^44^. Sedimentological studies correlate with the environmental proxies suggesting severe conditions^80^ (Table 4).

Lezetxiki in Arrasate, Gipuzkoa, was excavated by J. M Barandiarán between 1956 and 1968 and since 1996 has been excavated by A. Arrizabalaga. The site contains several levels corresponding to the Middle Palaeolithic from some of which a *Homo heidelbergensis* humerus and two Neanderthal teeth were recovered^81–83^. Mousterian Levels IV and V were sampled to provide an indication of the environments during that period. Collagen preservation was poor with these levels and samples did not yield sufficient collagen for radiocarbon dating and isotopic analysis, and will not be discussed further, but this serves to provide caution to other researchers contemplating analysis of remains from this site. Besides, Aurignacian Level IIIa, from the original excavation, was not sampled due to its possible mixing with Mousterian Level IIIb^34^, in addition to it having a possible Solutrean component.

### Materials

Bones of red deer (*Cervus elaphus*) and horse (*Equus* sp.), two of the most common mammal species represented during the regional Middle and Early Upper Palaeolithic^43,84–86^, were sampled. These specimens were selected strategically to measure the impact of broader climate on the isotopic values of two ecologically different species; grazers (horse) and intermediate feeders (red deer) to be observed. No contemporary browsers were available for comparison as they are scarcely represented during the period of study in the region. Specimens with evidence of anthropogenic modification (i.e., cut marks and/or anthropogenic breakage), associated with stone tools were targeted. Fused long bones of mature individuals were selected to prevent analysis of juveniles that might have prevailing weaning signatures^87,88^. In total, 138 animal individuals belonging to Mousterian (n = 61), Châtelperronian (n = 11), Aurignacian (n = 31) and Gravettian (n = 35) levels at six archaeological sites were analysed for δ^13^C and δ^15^N and 30 of these specimens were also analysed for δ^34^S (Table 1). The 12 horse and 18 red deer specimens selected for δ^34^S all contained >5 mg of collagen required for analysis and had δ^15^N values from the lower, middle and upper ranges of the dataset to explore the relationship between δ^15^N values and feeding locations. At the beginning of this project, radiocarbon dating using the AMS ultrafiltration method was undertaken to confirm the chronological attribution of the levels sampled^33^. Although some of the archaeological sites already had radiocarbon dates (mostly completed in the 1990s and early 2000s), new dates were run with ultrafiltration method (Table 2), which has greatly improved the reliability of the ages obtained due to the more effective removal of low molecular weight contaminants^89,90^, especially during this period of study.

## Results

### Collagen preservation and sample integrity

Collagen preservation within the >30ka fraction was excellent. Of the 138 archaeological specimens analysed, 124 provided sufficient collagen for analysis (82 red deer and 42 horse). Values discussed within this paper had C: N values between 2.9 and 3.6, suggesting *in vivo* collagen^91^, with 121 of these complying with the more rigid criteria of 2.9–3.4^92^. For δ^34^S analysis, all specimens had C:S collagen values between 600 ± 300 and atomic N:S collagen values between 200 ± 10^93^. Raw data, quality indicators and information for the samples that failed are provided within Supplementary Information Table 1.

### Results of the δ^13^C and δ^15^N analyses

#### Broad temporal trends

The δ^15^N values of both species range between 1.3‰ and 9.2‰, which is higher than typically observed for animals feeding within the same trophic level in the same geographical location^94^. The δ^13^C values range between −21.6‰ and −19.2‰. To explore wider broader environmental trends, specimens from each cultural period, defined by archaeological assemblage characteristics were grouped and populations were compared statistically, as outlined in the methods section at the end of this article.

**Figure 2:**
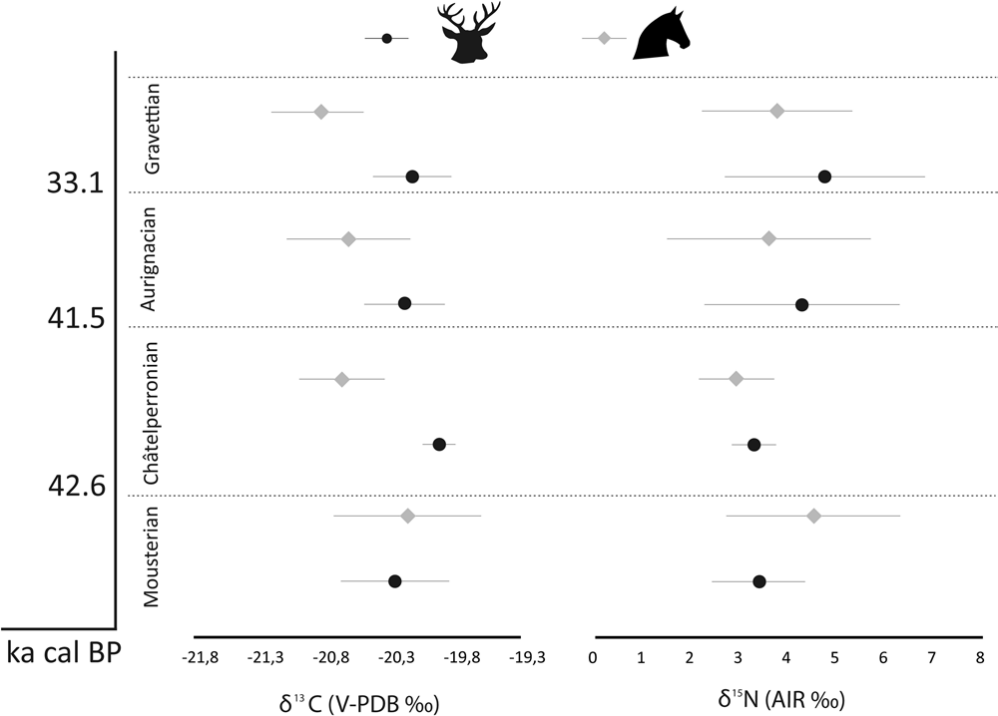
Mean red deer (denoted by black circles) and horse (denoted by grey diamonds) values with error bars showing 1σ for each cultural period. The chronological data are from Marín-Arroyo *et al*.^33^ which states that the Mousterian disappeared in the region by 47.9–45.1ka cal BP, while the Châtelperronian lasted between 42.6k and 41.5ka cal BP and the Mousterian and Châtelperronian did not overlap. The Aurignacian appears between 43.3–40.5ka cal BP overlapping with the Châtelperronian and ended around 34.6–33.1ka cal BP, after the Gravettian had already been established in the region.

**Figure 3:**
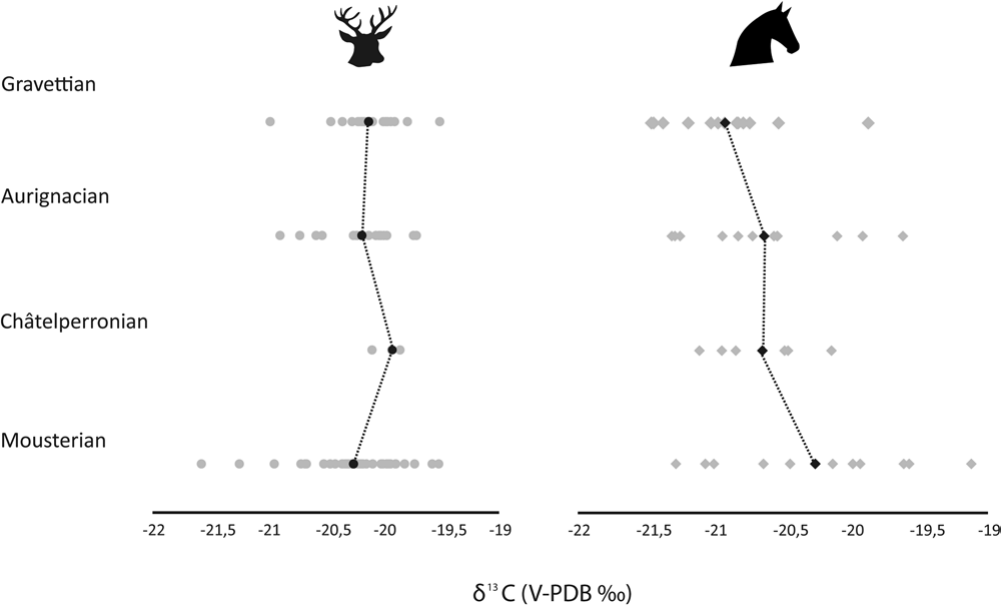
Mean and individual red deer (left) and horse (right) δ^13^C values plotted for each cultural period. Circles denote red deer, diamonds denote horses. Mean values are displayed in black and individuals sampled are displayed in grey.

**Figure 4:**
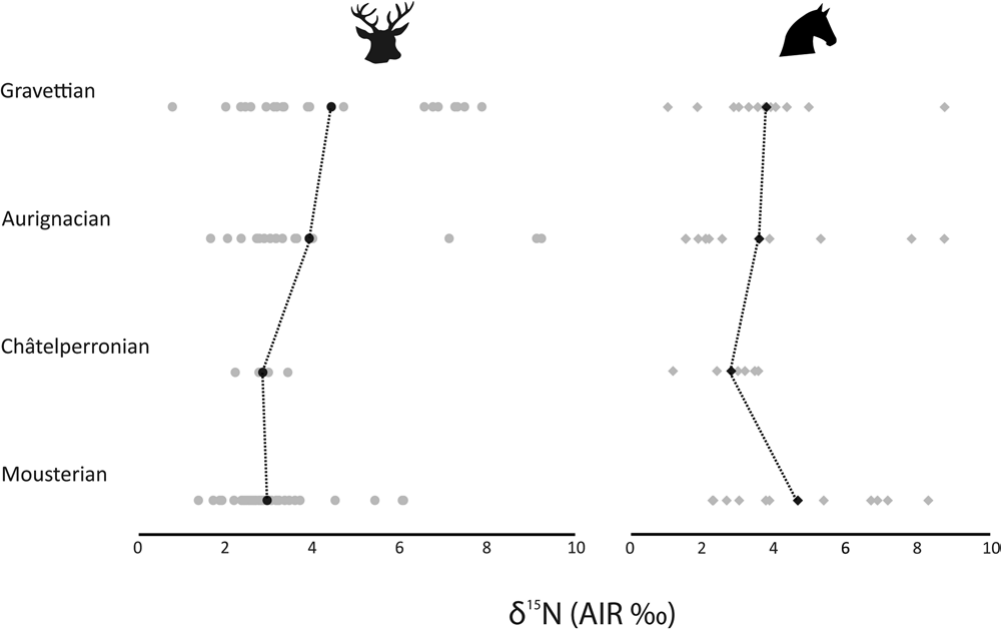
Mean and individual red deer (left) and horse (right) δ^15^N values plotted for each cultural period. Circles denote red deer, diamonds denote horses. Mean values are displayed in black and individuals sampled are displayed in grey.

**Figure 5:**
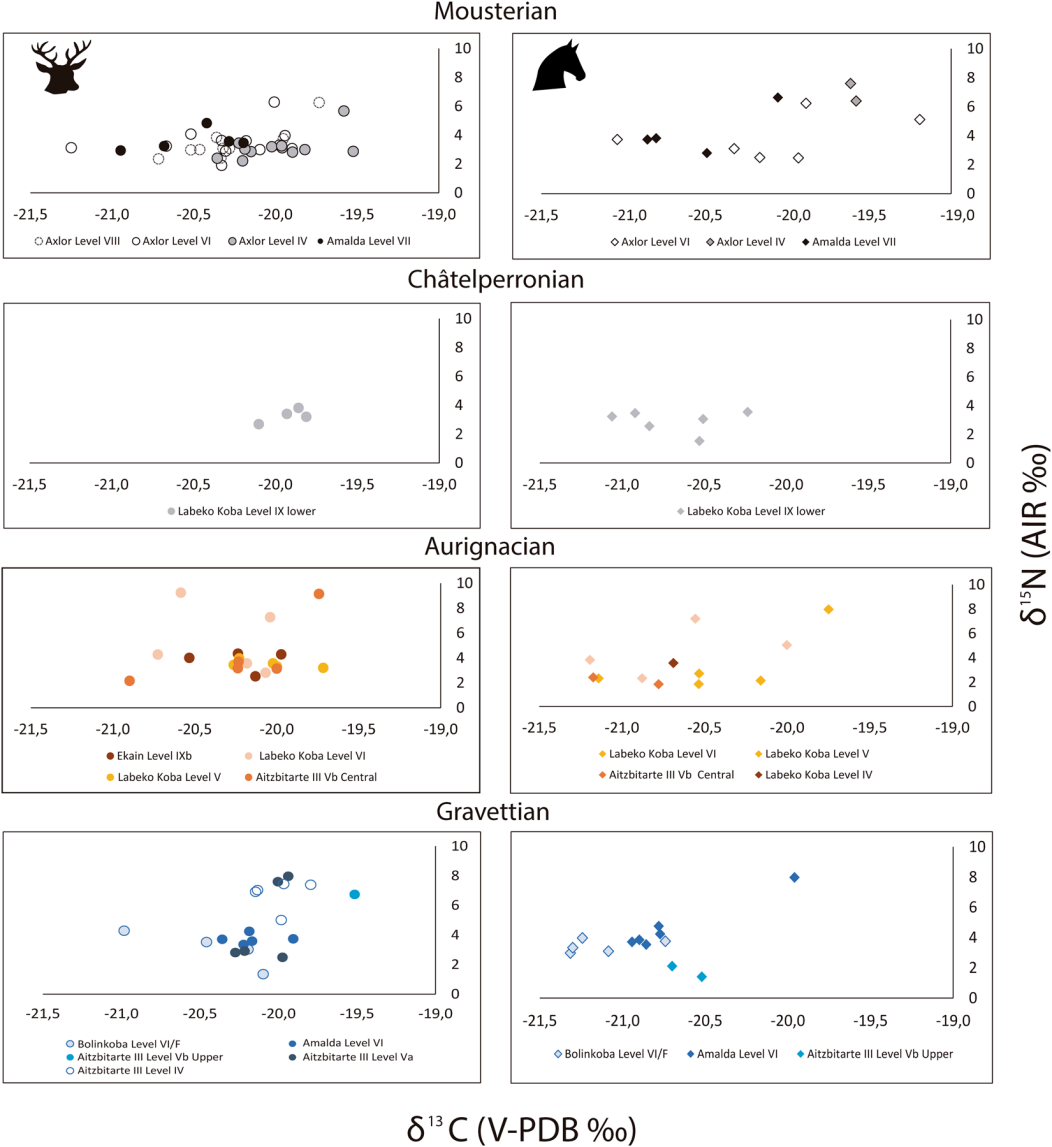
Bone collagen δ^13^C and δ^15^N values of red deer and horse specimens from the sites analysed in this study, for each cultural period and archaeological level. Red deer values are plotted on the graphs to the left (denoted as circles) and horse ones to the right (denoted as diamonds).

There is an increase in mean red deer δ^15^N values from the Mousterian (3.4‰) and Châtelperronian (3.3‰) to the Aurignacian (4.3‰) and Gravettian (4.7‰), although these means are likely influenced by the presence of individuals with elevated δ^15^N values (Table 5; Figs 2–5). The Mousterian red deer populations were statistically significantly different from those in the Aurignacian (*p* = 0.02) and Gravettian (*p* = 0.01) (Table 6). For the horse, the highest mean δ^15^N values are in the Mousterian (4.5‰), decrease in the Châtelperronian (2.9‰) and stay at a lower level throughout the Aurignacian (3.6‰) and Gravettian (3.8‰), but no statistically significant differences between δ^15^N values of horse populations is observed between any of these cultural levels (Table 5).

**Table 5:** Summary statistics for red deer and horse specimens for each cultural period analysed.

Cultural attribution	N	Red deer δ^13^C				Red deer δ^15^N			
		Mean	Min	Max	1σ	Mean	Min	Max	1σ
Mousterian	39	−20.3	−21.6	−19.5	0.4	3.4	1.9	6.3	1.0
Châtelperronian	4	−19.9	−20.1	−19.8	0.1	3.3	2.7	3.8	0.5
Aurignacian	19	−20.2	−20.9	−19.7	0.3	4.3	2.2	9.2	2.0
Gravettian	20	−20.1	−21.0	−19.5	0.3	4.7	1.3	8.0	2.1
		**Horse δ** ^**13**^ **C**				**Horse δ** ^**15**^ **N**			
Mousterian	12	−20.2	−21.0	−19.2	0.6	4.5	2.5	7.6	1.8
Châtelperronian	6	−20.7	−21.1	−20.2	0.3	2.9	1.5	3.6	0.8
Aurignacian	11	−20.6	−21.2	−19.7	0.5	3.6	1.8	8.0	2.1
Gravettian	13	−20.9	−21.3	−20.0	0.4	3.8	1.4	8.0	1.6

**Table 6:** Statistical test results comparing red deer δ^15^N and δ^13^C values within each cultural period. Statistically significant *p* values are highlighted in bold.

		Statistical comparisons of red deer δ^15^N values (*p* values)			
		Mousterian	Châtelperronian	Aurignacian	Gravettian
Statistical comparisons of red deer δ^13^C values (*p* values)	Mousterian	—	0.71	**0.02**	**0.01**
	Châtelperronian	**0.04**	—	0.33	0.20
	Aurignacian	0.50	0.05	—	0.51
	Gravettian	0.17	0.08	0.57	—

There is little temporal change in δ^13^C values. In the Châtelperronian, red deer have lower δ^13^C values than in the Mousterian (Table 5) and the populations were statistically different (p = 0.04) (Table 6). Horses had slightly lower δ^13^C values in the Châtelperronian compared to the Mousterian, but no statistically significant differences between populations within consecutive temporal periods were observed (Table 7).

**Table 7:** Statistical test results comparing horse δ^15^N and δ^13^C values within each cultural period. Statistically significant *p* values are highlighted in bold.

		Statistical comparisons of horse δ^15^N values (*p* values)			
		Mousterian	Châtelperronian	Aurignacian	Gravettian
Statistical comparisons of horse δ^13^C values (*p* values)	Mousterian	—	0.09	0.09	0.53
	Châtelperronian	0.07	—	0.93	0.15
	Aurignacian	0.06	0.96	—	0.38
	Gravettian	**0.00**	0.27	0.17	—

When comparing the red deer and horse values, both species have similar mean δ^13^C values in the Mousterian and horses have a mean δ^15^N value 1‰ higher than the red deer (Fig. 2). In the Châtelperronian, Aurignacian and Gravettian, there is a shift in the diet of horses relative to the red deer and the mean horse values are lower in δ^13^C and δ^15^N values than red deer ones (Table 5) (Fig. 2).

### Period-specific trends

#### Mousterian

Within the Mousterian, both the red deer and the horses display wide ranges in the δ^15^N and δ^13^C values observed. A group of horses and red deer have δ^15^N values between 1.8‰ and 5.1‰. There is another cluster of horse and red deer specimens with δ^15^N values within ± 1σ of the mean, ranging between 5.7‰ and 9.5‰ (Figs 2, 4), which have been identified as belonging to different groups using cluster analysis (horse cophenetic correlation coefficient = 0.91; red deer = 0.87) The latter is outside of the range typically expected for individuals feeding within the same geographical location^94^. This pattern is particularly observed in Mousterian Levels IV, VI, VIII of Axlor and in Amalda Level VII. There is little difference in the δ^13^C values of either species and a clear overlap is seen (Figs 3, 5).

#### Châtelperronian

Level IX lower at Labeko Koba is the unique Châtelperronian level in the eastern Cantabrian region dated by ultrafiltration^35^. All horses and red deer plot within the same δ^15^N trophic range (horse: 1.5–3.6‰, red deer: 2.7–3.8‰) (Figs 4, 5.). There is no overlap between the two species in the δ^13^C values (red deer min −20.1‰, max −19.8‰, horse min −21.0‰, max −20.2‰). Red deer δ^13^C values are consistently higher relative to the horse analysed in this level and difference in δ^13^C values between the two populations is statistically significant (*p* = 0.01) (Table 8).

**Table 8:** Statistical test results comparing red deer and horse δ^15^N and δ^13^C values within each cultural period. Statistically significant *p* values are highlighted in bold.

			Statistical comparisons of red deer and horse δ^15^N values (*p* values)							
			Mousterian		Châtelperronian		Aurignacian		Gravettian	
			Red deer	Horse	Red deer	Horse	Red deer	Horse	Red deer	Horse
Statistical comparisons of red deer and horse δ^13^C values (*p* values)	Mousterian	Red deer		0.06	0.71	0.66	**0.02**	0.33	**0.01**	0.13
		Horse	0.60		0.39	0.09	0.76	0.09	0.68	0.53
	Châtelperronian	Red deer	**0.04**	0.39		0.52	0.33	0.58	0.20	0.43
		Horse	**0.01**	0.07	**0.01**		0.06	0.93	**0.05**	0.15
	Aurignacian	Red deer	0.50	0.85	0.05	**0.01**		0.11	0.51	0.66
		Horse	**0.01**	0.06	**0.02**	0.96	**0.01**		0.07	0.38
	Gravettian	Red deer	0.18	0.91	0.08	**0.00**	0.57	**0.00**		0.36
		Horse	**0.00**	**0.00**	**0.01**	0.27	**0.00**	0.17	**0.00**	

#### Aurignacian

Aurignacian Levels VI and V in Labeko Koba and Level Vb Central of Aitzbitarte III, exhibit a similar pattern to the Mousterian, with a group of individuals with lower δ^15^N values (range; 2.3‰ to 5.0‰) and another with elevated δ^15^N values (range; 6.7‰ to 9.2‰) (Fig. 4), and cluster analysis showed these individuals as belonging to different groups (horse cophenetic correlation coefficient = 0.93; red deer = 0.95). Within Level V at Labeko Koba all individuals analysed plot within the lower nitrogen range, except for one horse and Level VI contains two red deer with elevated δ^15^N values (Fig. 5). The δ^13^C ranges of the individuals with high and lower δ^15^N values fall within the same range (−21.2‰ to −20.5‰, Fig. 3) and there is a clear differentiation in the δ^13^C values of horse and red deer, with horse having consistently lower values (Fig. 2).

#### Gravettian

In the Gravettian horse and red deer are found to have lower δ^15^N values (range 1.3‰−5.1‰), apart from a group of red deer with higher values (6.9‰−9.5‰) observed within Aitzbitarte III Level IV, Amalda Level VI and at Bolinkoba Level VI(F) (Figs 4, 5). The red deer and horses with higher δ^15^N values from this period were identified using cluster analysis as belonging to a different group (horse cophenetic correlation coefficient = 0.94; red deer = 0.91). This is consistent in the preceding periods, although, unlike the earlier periods, no horses show higher values. As seen in the Châtelperronian and Aurignacian, horses have lower δ^13^C values than red deer (Fig. 2).

### Results of the δ^34^S analysis

**Figure 6:**
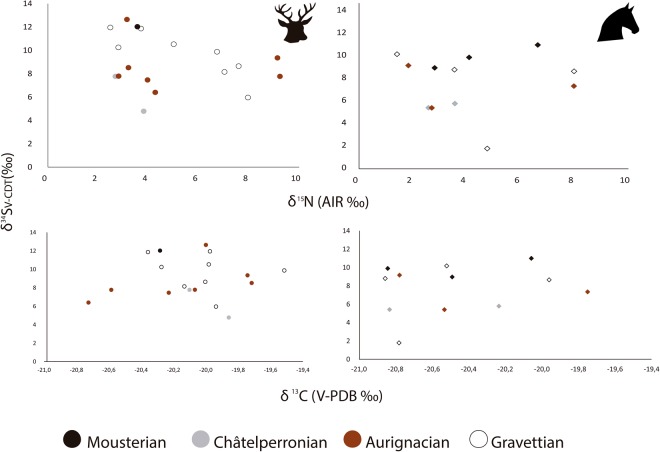
Bone collagen δ^34^S values plotted with the δ^15^N values (above) and δ^34^S values and δ^13^C values (below) for each of the cultural periods sampled within the eastern Cantabrian region. Red deer values are plotted in the graphs on the left (denoted as circles) and horse values are plotted on the right (denoted as diamonds).

The δ^34^S values of the red deer and horse analysed ranged between 1.8‰ and 12.6‰, with most specimens falling between 5‰ and 11‰ and are consistent with animals inhabiting terrestrial ecosystems^20^. There is no linear correlation between δ^34^S and δ^15^N values (r = 0.049) or δ^34^S and δ^13^C values (r = 0.076), which would be expected if there was a link between the sulphur isozones and the carbon/nitrogen zones being exploited (Fig. 6) (Supplementary Information Table 2), and no clear groupings of values were identified using cluster analysis. Sulphur values within local food webs are controlled by the bedrock composition, atmospheric deposition and microbial processes^95^, rather than by dietary behaviour, enabling both herbivore species to be directly compared.

Within Châtelperronian Level IX Lower at Labeko Koba, the δ^34^S values range between 4.8‰ and 7.8‰. At Labeko Koba, within Level V, values ranged between 5.4 and 8.5‰ and for Level VI individuals have values between 6.4‰ and 7.8‰.

**Figure 7:**
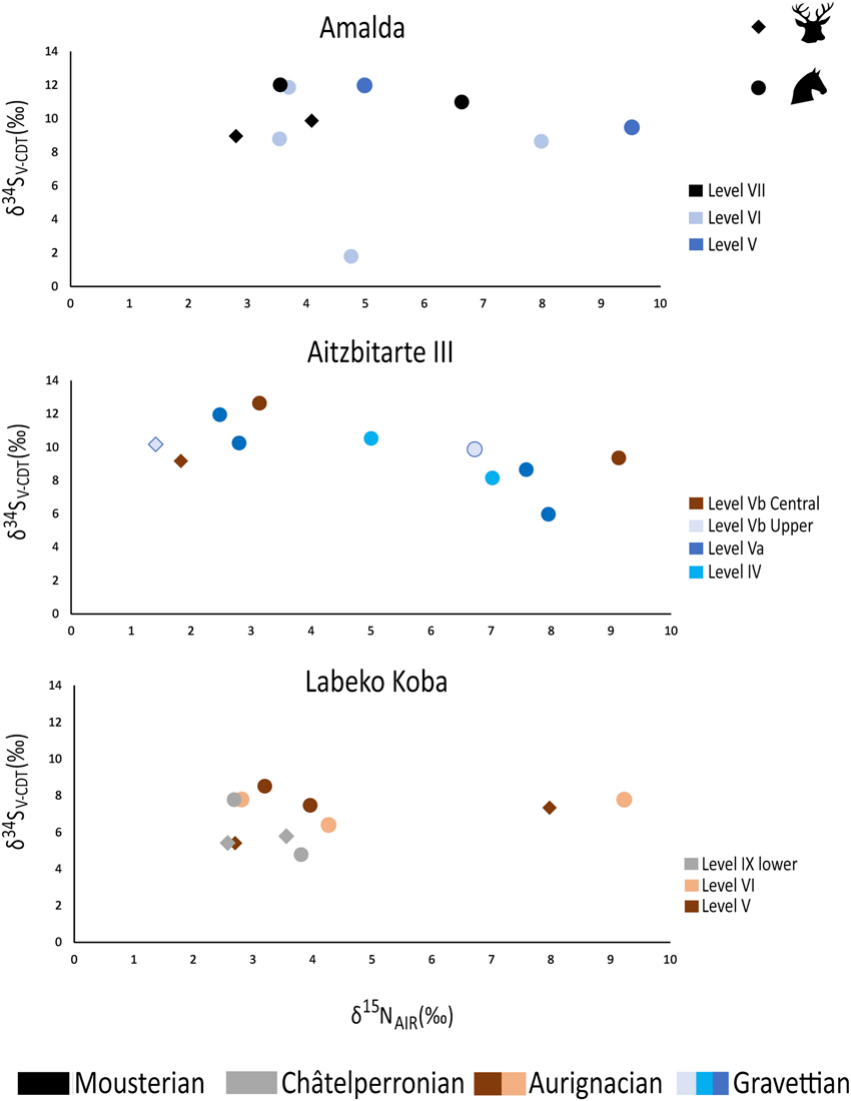
Red deer and horse δ^34^S values plotted with the δ^15^N ones for Aitzbitarte III, Labeko Koba and Amalda showing the distribution of values for each archaeological level and cultural period. Red deer are plotted as circles and horses are plotted as diamonds.

In Amalda the values were generally higher than at Labeko Koba. In Amalda Level VII, δ^34^S values ranged between 9‰ and 12‰ and in Amalda Level VI, the δ^34^S values from the bulk of the individuals ranged between 8.7‰ and 11.9‰ (Fig. 7), with only one specimen showing an uncharacteristically low δ^34^S value of 1.8‰ with a δ^15^N value of 4.8‰ (Fig. 7). This was inconsistent with any of the other specimens analysed in this study.

At Aitzbitarte III, δ^34^S values ranged between 6‰ and 12.6‰ (Fig. 7). The Evolved Aurignacian samples in Level Vb Central ranged between δ^34^S values of 9.4‰ and 12.6‰. For the Gravettian at the site, Level Va one individual had a lower δ^34^S value of 6‰ with a δ^15^N value of 8‰. All other specimens from Levels Va, IV and Vb Upper fall within a δ^34^S range of 8.2‰−11.9‰.

## Discussion

### Broad trends in bone collagen values and its impact for environmental reconstruction

Within the red deer and horses sampled no δ^13^C value is higher than −22.5‰, meaning that they are consistent with the consumption of C3 plants in an open landscape, according to Drucker *et al*.^24^. They are also stable through time, indicating that there was little change in tree cover throughout the period of study (i.e., late MIS3). The higher δ^13^C values of horses, in comparison to red deer, observed in the Mousterian, and an opposite trend afterwards suggest a change in environment in the temporal sequence related to the horse niche. Typically horses persistently select poor quality, low-protein, high-fibre browse a fact which results in a relative depletion in the δ^15^N and δ^13^C values^96^, as seen in this study in the Châtelperronian onwards. A similar pattern has been observed in other Palaeolithic faunas in Central Europe, where horses are consistently depleted in ^13^C and ^15^N relative to red deer due to this dietary preference^21,22,97–99^. This change in the horse relative δ^13^C values demonstrate a shift in the suite of vegetation, resulting in niche separation of red deer and horses, likely expressing environmental shifts, resulting from wider climatic changes between the Mousterian and the later periods studied. Other palaeoenvironmental records in the region during this period of study are sparse (Table 4). The only Mousterian level with pollen information is Amalda VII, and it is mainly represented by *Pinus* and the *Cichorioideae*, although pollen preservation at the site was poor and pollen samples were selected from an area of the site with possible disturbance. During the Châtelperronian (represented by Level IX Lower at Labeko Koba) pollen evidence indicates that steppic vegetation dominated (Table 4). Similarly, trends based on the data from the sites with Aurignacian and Gravettian levels indicate that low tree cover, and steppic vegetation, such as the *Poaceae/Gramineae* grasses *Cedrela tubiflora* and *Compositae liguliflor*, dominated the landscape (Table 4). The shift towards more open, steppic landscapes, after the end of the Mousterian, environments more favourable to the niche of horse, may explain this change in the diet of horses, although its representation in the macromammal assemblages remained stable through the period of study in the eastern Cantabrian region (Table 3) and could indicate a climatic change at this time.

For the δ^15^N values, the presence of individuals with elevated values during the Mousterian, Aurignacian and Gravettian prevents broad patterns from being clearly observed. In the neighbouring region of SW France, a population level increase in δ^15^N values of between 2‰ and 4‰ during the Aurignacian was identified at around 31–35 ka uncal BP, being interpreted as a climatic change in the Early Aurignacian, potentially relating to episodes of aridity^21^.

### Larger ranges in δ^15^N values in the Cantabrian Region

The wide inter-individual nitrogen ranges observed within archaeological levels during the Mousterian, Aurignacian and Gravettian levels established in both the red deer and horse requires further exploration. It is not seen during Châtelperronian, but this may be a product of the small sample, consisting of only one archaeological level from one site.

Typically differences between herbivorous individuals of up to 2‰ in δ^15^N are expected on an intra-site level^94^. In this study, however, the dataset shows a δ^15^N value range of 5‰ and above for both species. Furthermore, the site of Antoliñako Koba, also in Gipuzkoa, exhibited a similar phenomenon to the current study with six red deer dating to the Gravettian period having δ^15^N values greater than 7‰ (maximum value 8.1‰, range 4.8‰, σ = 1.8, Table 6 in^100^), alongside a group of five individuals with lower values (3–5‰)^97^. Comparative red deer datasets of archaeological specimens, predominantly from the Late Upper Palaeolithic, all fall within lower δ^15^N ranges (typically between 3 and 5‰) with smaller standard deviations (Table 6)^100^.

The causes of elevated δ^15^N values in bone collagen can be related to either physiological or environmental factors. Regarding physiology, juvenile animals being nursed produce a δ^15^N value around one trophic level above that of their mothers^87,88,101^. However, all individuals analysed in this study were archaeozoologically determined to be adults, based on bone fusion, so this factor can be disregarded. Short-term stress episodes such as starvation or pregnancy can impact on δ^15^N values within an individual^102,103^, although to register in the long-term bone collagen signature, this had to have been experienced over long durations. Physiological factors are not sufficient to explain the pattern.

In environmental terms, altitude has been observed to have both positive^104,105^ and negative effects^106^ on δ^15^N values of plants and animal tissues. Positive correlations between plant δ^15^N values and altitude, related to soil activity and temperature differences, could exist, although a change in δ^13^C might also be expected. Altitudinal differences of up to 1,000–4,000 m are needed to have a substantial impact on isotopic values in plants^104,107^. Altitudes of up to 2,000 m in the Cantabrian Cordillera exist, but only in the west^31^, although none of the sites in our sample are located over 350 m.a.s.l. Additionally, feeding habits at high altitudes are not consistent with the ecology of horse and red deer, which typically do not inhabit higher elevations for prolonged periods of time. Modern ecological studies of red deer populations inhabiting mountainous regions demonstrate that red deer typically do not reach altitudes above 500 m^108^, although caution must be considered when using modern ecological analogues. Nonetheless altitude differences are not sufficient to explain the large differences in δ^15^N values seen within this dataset. Another environmental factor is the difference in the parts of plants consumed by the animals. An increase in leaf consumption or a shift from eating shrubs or trees to grasses could both explain the increased δ^15^N values^109,110^. Vegetation change was cited as an explanation for increased ranges in δ^15^N values of red deer in the Jura uplands of eastern France during the Late Glacial period^23^. If regular consumption of different types of vegetation by individuals could cause differences in the δ^15^N values, we would expect this to typically affect the δ^13^C values as well^111^ which is not observed in this study, meaning that other environmental factors must be at play.

Differing mycorrhiza in ecosystems depending on localised conditions can impact on ^15^N concentrations absorbed by plants^112,113^. Soil activity of nitrogen fixing mycorrhizal is reduced within colder environments and consequently, a temperature decrease is observed^114,115^, and decreased δ^15^N is often associated with lower mean annual temperatures^116^. Soil nitrogen content within different floodplains can also vary depending on water table height^117^, impacting on plants growing in different valley systems. Additionally, several worldwide studies demonstrate a negative correlation between plant δ^15^N values and precipitation^116,118–120^. Increased aridity due to reduced precipitation can produce ^15^N-enriched values^105,121–124^. Consequently, animals habitually feeding in drier environments would be expected to have higher δ^15^N values than those consistently feeding in wetter environments.

Given the above, to explain differing δ^15^N values in bone collagen within individuals in the same archaeological level, there are two plausible scenarios. Firstly, it could be that animals brought to the caves were being hunted in two isotopically distinct environments (‘isozones’), producing a mixed assemblage of animals with different isotopic signatures within the same archaeological levels, as has been recently proposed for contemporary regional sites in the central area of the Cantabrian region^125^. At early times in site occupation, humans might have consumed “trail food”—joints of transported meat acquired in their area of hunting—before they acquired local game. It is conceivable that human groups moved across the low Basque Mountains between the upper Ebro drainage and the Cantabrian coastal region as part of seasonal rounds (as suggested by the presence of Trans-Cordilleran flints (Urbasa, Treviño, etc.) in the sites of Bizkaia and Gipuzkoa. Secondly, it is conceivable that environmental fluctuations occurred during the formation of the archaeological levels, with changing environmental conditions of temperature and aridity, causing the accumulation of individual animals with distinctly different stable isotope signatures, as proposed by Bocherens *et al*.^21^ for the ungulate assemblage studied in the SW France during the Middle to Upper Palaeolithic transition. The long-time formation of the archaeological levels analysed converted them into thick palimpsests which could represent multiple human occupations perhaps over centuries, and this fact might also explain the changes in the observed climatic conditions.

Regarding the first hypothesis, the δ^15^N results could be a result of animals feeding in two isotopically distinct territories, with human activity being responsible for bringing these two groups of animals habitually feeding within different geographical regions: one, with lower δ^15^N values and another, with higher δ^15^N values. Animals may have been procured from different valley systems, which may have differing soil δ^15^N values due to mycorrhiza^112,113^ or with varying baseline nitrogen values related to water table height^117^. The lithology in the region is highly varied, with Jurassic and Cretaceous rocks predominating in the coastal regions of the Basque country, interspersed with pockets of Triassic clays, gypsum and seams of tertiary rocks within the Ebro basin and further south, comprising predominantly of Tertiary rocks^126,127^. This high diversity in rock types, coupled with distinctive topographical conditions, could support the existence of micro-environments within the region, as seen in the province of Cantabria to the West^125^. The specimens from Antoliñako Koba in Bizkaia, showed a similar scenario of higher and lower ungulates δ^15^N values during the Gravettian^100^ (Table 9). The explanation provided was that those animals might have been obtained from different hunting locations. Lower δ^15^N values are seen during the Châtelperronian of Labeko Koba (Level IX lower), where ungulates brought to the site by carnivores and alter scavenged by humans, are indicative of a local δ^15^N signature at that time. Another possibility is that the outlying individuals came from a drier region, such as the area to the south of the Cantabrian cordillera, potentially the Llanada Alavesa in Alava, Province of the Basque Autonomous Community (Fig. 1), in the rain shadow of the mountains and outside the reach of the Foehn effect. Proposed hunting ranges during the Middle and Upper Palaeolithic, based on the Optimal Foraging Theory, suggest that hunting territories were modified to target higher ranked prey, with movements between the coast and the mountains^86^, although the mobility in this particular region based on predictive models to calculate the potential distribution of ungulates, according to topography and related vegetation, indicate an area of exploitation within the two-hour walking territories of the sites^128^. At Axlor and Amalda studies suggest that, due to the steep topography surrounding these sites, occupants would have had extended territories to target specially selected prey^128^. Based on this, hunting in various locations within the wider sites was an option as reveal by the catchment territory of lithic raw materials in the sites analysed^38^. Thus, macromammal assemblages from the studied sites represent taxa related to the topographic location of each site, with high representation of montane (or at least, steep, rock environment) ungulates, fluvial valleys and plains or all of the above. In the sites studied, red deer is the most common taxon (24%), followed by bovines (18%), horses (7%), Spanish ibex (7%) (Table 3) and exceptionally, chamois (at most 42%) is highly represented in Amalda Levels VI-VII attributed by one researcher to a carnivore accumulation with sporadically human occupations^40,42^. Taphonomic analyses in Aitzbitarte III show that prey transport was mostly dependent on body size. Age profiles indicate exploitation of prime-age individuals, although infantile and juveniles are also represented^43^. Bone marrow extraction has been documented in all the sites, in combination with butchering marks on the ungulates and even on some carnivores^41,42^. For instance, in Aitzbitarte III Level Vb, cut marks were identified in an ulna of cave bear and, in Axlor exploitation of carnivores (wolf and dhole) and birds (raven and golden eagle) by Neanderthals were also documented^39^.

**Table 9:** Red deer comparisons of mean, minimum, maximum and range δ^15^N values from existing isotopic studies in the Cantabrian Region. No horse specimens were available as a comparison.

Province	Eastern Cantabria Region Middle Pal and Early Upper Pal. (This Study)	El Castillo Cave Middle Pal and Early Upper Pal^121^	Covalejos Cave Middle Pal and Early Upper Pal^121^	Kiputz IV Paleontological site (~25-13 ka cal BP)^143^	Antoliña Koba Upper Pal (~36–12 ka cal BP)^97^	El Mirón Late Upper Palaeolithic^30^	La Paloma (Late Upper Pal.)^144^
	Gipuzkoa and Bizkaia	Cantabria	Cantabria	Gipuzkoa	Bizkaia	Cantabria	Asturias
n=	88	45	33	29	28	127	18
Mean	4.0	3.8	4.4	3.7	4.7	2.9	4.9
Minimum	1.3	1.0	1.4	2.4	3.4	0.6	3.9
Max	9.2	5.2	7.9	5.5	8.1	4.5	5.9
Range	7.9	4.2	6.5	3.1	4.8	3.9	2.1
1σ	1.6	0.9	1.4	0.9	1.8	0.6	0.6

To evaluate animal provenance further, δ^34^S analyses was undertaken. Sulphur studies of modern ecosystems note a 1.9‰ difference within ungulate populations, with higher variability in archaeological studies of 2.4‰ variation observed^20^. At Labeko Koba (Level IX lower) a 3‰ difference was observed between specimens, all of which resulted from a hyena-formed deposit, later scavenged by humans^68^ and can be assumed to represent animals accumulated from the local area. For the other sites studied, excluding outliers, a range of 4‰ was observed. This suggests that the level of variation for animals feeding within the same region could be up to 3-4‰, particularly in landscapes within deep, narrow, sinuous valleys. The δ^34^S values from all levels at Amalda and Aitzbitarte III were typically higher than those seen within the levels analysed at Labeko Koba and may not have derived from the same locality. All three sites are in different, but adjacent valleys, which may explain the differences in baseline δ^34^S values observed between them (Fig. 7). One red deer from Amalda Level VI and one from Aitzbitarte III Level Va had very low δ^34^S values in comparisons to other analysed from the same levels, suggesting procurement of animals from at least two locations by the inhabitants of these sites (Fig. 7).

There was no linear correlation between δ^15^N and δ^34^S values, suggesting that if the animals with higher δ^15^N were being hunted from elsewhere, they were not necessarily coming from a different sulphur isoscape. Considering that ^34^S systems are dictated by geographically determined factors, such as rock type and precipitation^20,95^ and δ^15^N systems are controlled by more biological factors, such as nitrogen fixation by plants, nitrogen and sulphur isoscapes may not necessarily be the same.

The hypothesis of multiple catchment territories with different geographical and environmental conditions producing distinctive isotopic values (i.e. from the Llanada Alavesa) could be considered less feasible, when contemplating hunting efficiency for these hunter-gatherer groups in relation to the distance to the camp site and prey size hunted, among other factors^129^.

Regarding the second hypothesis, the high variability of δ^15^N values of the red deer and horses within the same archaeological levels could be a product of cycling environmental conditions within these archaeological levels (accumulated usually during multiple human occupations along hundreds of years and sometimes, thousands), potentially relating to fluctuating temperatures and cycling periods of aridity. The second part of MIS3 was a time of remarkable instability with abrupt and rapid climatic changes. The Heinrich events produced a sequence of cooling and warming cycles^2^ and the Dansgaard-Oeschger cycles presented periods of climatic fluctuation^11^, occurring sporadically within millennial scales^1,4,130^. Offshore records from the NW of the Iberian Peninsula exhibit cycles of wetter-drier periods^13^. Marine core MD95-2042, from the SW Margin of the Iberian Peninsula, demonstrated millennial scale climatic changes, ranging from temperate and humid to cold and dry continental conditions^130^. Available environmental proxies for these sites do not exhibit evidence of intra-level fluctuations (Table 4), although these indicators may not be sensitive enough to reflect these smaller changes on this temporal scale. Isotopic values obtained in this study provide a direct indication of past environment, directly related to evidence of human occupation.

In terms of chronology of the timescales of the levels involved with the fluctuating cycling climatic conditions, the Mousterian levels within Axlor date beyond the radiocarbon limit, and it is possible that these represent thick deposits spanning several millennia. For the other archaeological levels analysed, the chronologies represented are much tighter. At Aitzbitarte III Level Vb Upper to c. 32-31 ka BP and Level Vb Central dated to c. 31 ka BP, Level Va it is 31–26 ka BP and Level IV it is 29 ka BP. For Labeko Koba, the chronologies are equally well defined, with Level IV dating to 33.5 ka BP, Level V to 34.6 ka BP, Level VI to c. 35 ka BP and Level IX Lower to 38-37 ka BP^35^ (Table 2). The tight chronological resolution of these levels, means that environmental oscillations would had to have occurred in a timeframe of hundreds of years, and the abrupt nature of these changes could have implications for human subsistence and behaviour. Other contemporaneous European sites with analyses of both red deer and horses^21,23,131^ do not show evidence of rapid frequency of environmental shifts, a fact which may be related to site formation chronologies or local environmental conditions in the particular studied regions. Further exploration of this hypothesis is required, with more precise chronological timescale, in additional regions occupied by Neanderthals and AMH that represent similar timeframes, as presented here, would be beneficial in the future.

## Conclusions

The results of this study of bone collagen δ^13^C, δ^15^N and δ^34^S values conducted on animal remains, with evidence of human manipulation, pertaining to the Middle-Upper Palaeolithic transition in the Cantabrian Region of Northern Spain provide a human-related reconstruction of the past environmental conditions at the time the replacement of late Neanderthals by the anatomically modern human populations took part in the Atlantic zone of Iberia. During the Palaeolithic, the Cantabrian region was special in providing a variety of habitats to exploit for both human species, and whilst MIS3 was a period of climatic instability in Europe, this did not prevent either human populations from successfully occupying this region. The δ^13^C values results show that after the conclusion of the Mousterian, a shift in climate, was expressed in the eastern part of the Cantabrian region by a change in the dominant vegetation, generating more open landscapes, with the horse niche changing to favour the lower-quality browse that they preferentially consume, an hypothesis which is supported by the other environmental proxies including sedimentology, pollen and micromammals. The δ^15^N results show high inter-individual variability within the same archaeological levels in the Mousterian, Aurignacian and Gravettian. This could be linked to animals hunted in different territories, although whether this represents micro-environments within the eastern Cantabrian region, and the same valley system, as proposed for the central region of Cantabria or animal carcasses (or joints of meat) being brought from further afield as trail food, is difficult to determine. However, we do not discard the possibility that these large differences in δ^15^N values are more a reflection of changing environments during the formation of these archaeological levels, representing several generations of human occupation activity. If so, changing environmental conditions across the transition would have had implications for human evolution and adaptive skills. Further research is required to explore, involving high resolution sampling and dating to observe the difference variation in isotopic values among the animals consumed by human groups during the period of study in this region.

### Methods summary

Collagen extraction was undertaken following procedures outlined in Richards and Hedges^132^ with an ultra-filtration step^133^. Bone fragments between 0.5 and 0.8 g were cleaned using aluminium oxide air abrasion, before demineralisation in 0.5 M HCL at 6–8 °C for between 3–10 days,and were washed three times using de-ionised water. Samples were gelatinised in a weak acidic solution (pH3 HCL) at 70 °C for 48 hours, then filtered with 5–8 μm Ezee® filters, prior to ultrafiltration to separate out the larger >30ka collagen chains. The >30ka fraction was frozen and lyophilized for 48 hours. Collagen was analysed in duplicate, using a Delta XP mass spectrometer coupled to a Flash EA 2112 elemental analyser at the Department of Human Evolution, Max Planck Institute for Evolutionary Anthropology (Leipzig, Germany). The δ^13^C values and δ^15^N values are reported relative to the V-PDB and AIR standards. International and internal standards were used to calculate analytical error which was ± 0.1‰ (1σ) or better. The mean difference observed between duplicate measurements was 0.03 for δ^13^C, and 0.01 for δ^15^N. Sufficient collagen was not available for duplicate analysis for specimens: AXL01, LAB06, and EK02, EK04, EK05 and EK06. Analysis for δ^34^S values was undertaken at the University of British Columbia Stable isotope laboratory in Vancouver using a MicroCube IsoPrime 100 DI mass spectrometer.

Isotopic values were analysed statistically using a Mann-Whitney U test, with a post-hoc Holm-Bonferroni correction^134^. A *p* value of <0.05 or less was deemed to be indicative of a statistically significant result. To compare nitrogen value groupings within the dataset classical cluster analysis was used. All tests were undertaken using the statistical package PAST^135^.

A summary of environmental proxies at the sites investigated in this study, including pollen, microfauna, macrofauna and sedimentology, when available, have been included in the discussion to enhance interpretation of the results and provide general environmental context (Table 4).

## Electronic supplementary material

Supplementary Dataset 1
